# Supplementary material for: The Impact of Praise on Cooperative Behavior in Three-Player Public Goods Games and Its Gender Differences
Source: Behav Sci (Basel). 2024 Mar 22;14(4):264. doi: 10.3390/bs14040264 (PMC11047728; doi:10.3390/bs14040264)
Supplement: Supplementary file 1 [file behavsci-14-00264-s001.zip › behavsci-2886613-supplementary.pdf]

Experimental Flows Page by Page for Praise and Cooperation Study—praisePaper2

March 22, 2024

Contents

1 Public Praise Condition **2**

2 No Praise Condition **14**

1 Public Praise Condition

Page 1 Welcome Page

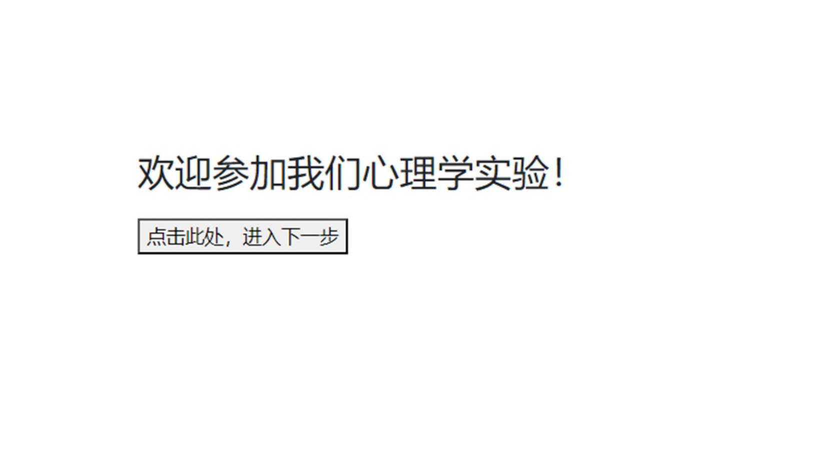

Welcome to attend our psychological experiment.
Click here to Next Page.

Page 2 Study Information Notice

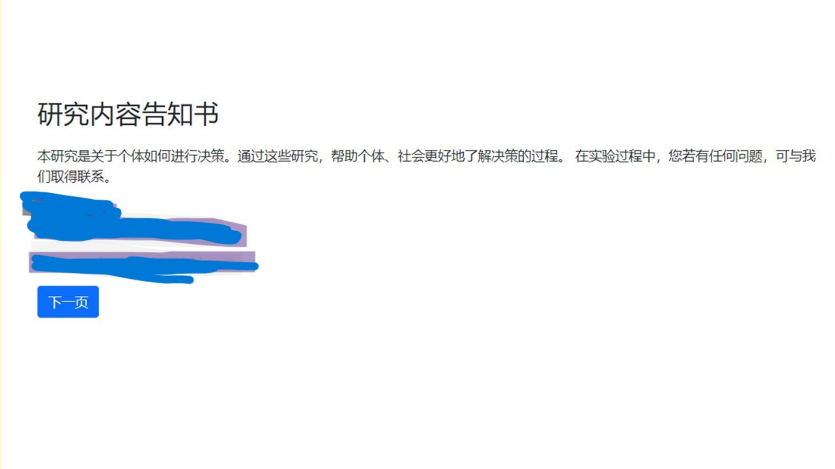

Study Information Notice

This study is about how to make decision. Through these studies, we are able to help individuals, society to understanding the process of decision making. During the progress of experiment, if you have any questions, please feel free to contact me.

Contact Information of Researchers. [cover for privacy issue.]

Page 3 Personal Information Collection

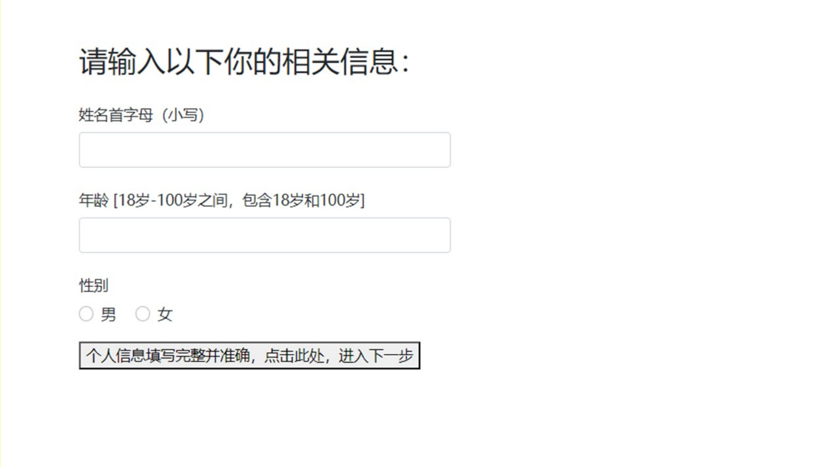

请输入以下你的相关信息：

姓名首字母（小写）

年龄 [18岁-100岁之间，包含18岁和100岁]

性别

男 女

个人信息填写完整并准确，点击此处，进入下一步

Your initial name (lower cases)

age: (18 to 100 years old)

gender: male; female

Click here to the next page.

Page 4 Experimental Instruction

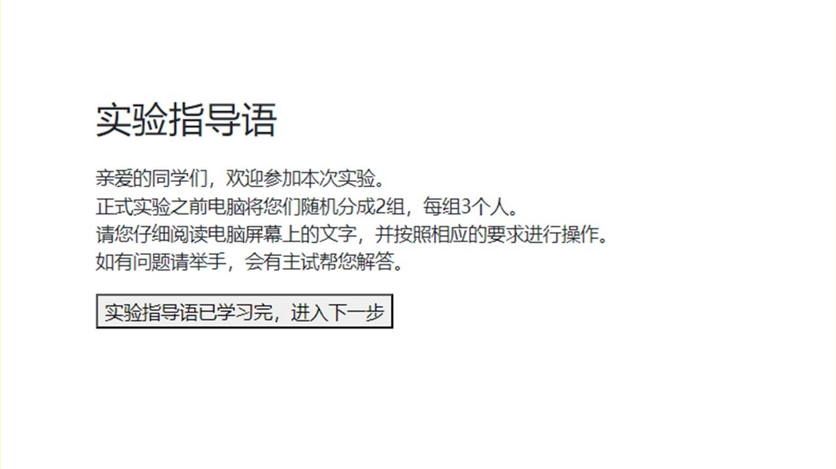

Experimental Instruction:

Welcome to our experiment. Before we start our experiment, you will be randomly into 2 groups, and each group will be assigned with three players. Please read carefully with the word in the screen, and operate according to the instruction. If you have any question, we will answer your question as detailed as we can.

Page 5 Instruction for the Game

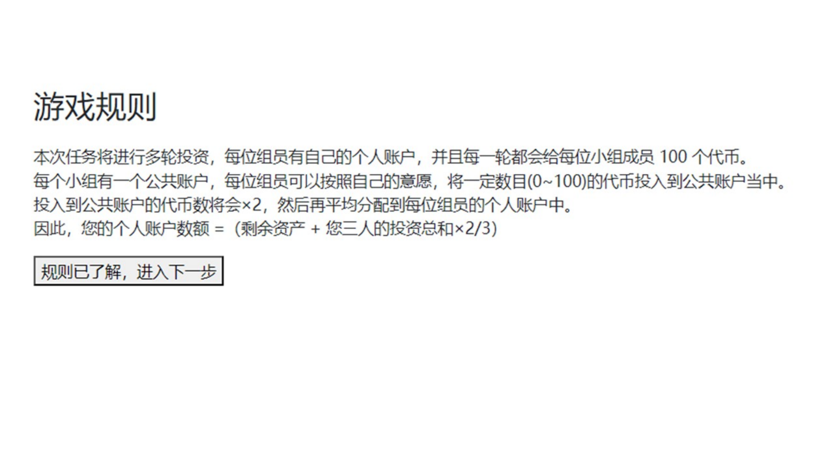

This task will involve multiple rounds of investment, with each team member having their own personal account, and each round, every team member will receive 100 tokens. Each group has a public account, and each member can invest a certain number X_i of tokens into the public account as they wish. ($0 <= X_i <= 100$) The tokens invested in the public account will be doubled and then evenly distributed to each team member's personal account. The final amount in each person's personal account is: $(100 - X_i) + (X_i + Y) \times 2/3$.
I have understood the instruction, click here to next move.

Page 6 Rule Comprehension Task

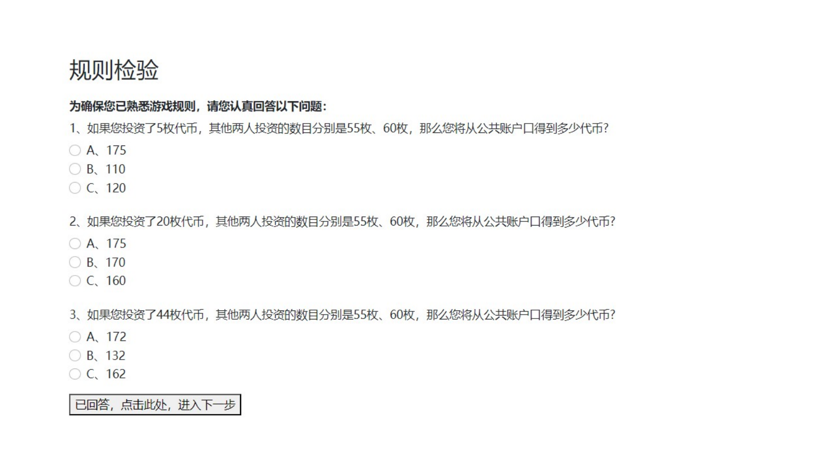

规则检验

为确保您已熟悉游戏规则，请您认真回答以下问题：

1、如果您投资了5枚代币，其他两人投资的数目分别是55枚、60枚，那么您将从公共账户口得到多少代币?

A、175
B、110
C、120

2、如果您投资了20枚代币，其他两人投资的数目分别是55枚、60枚，那么您将从公共账户口得到多少代币?

A、175
B、170
C、160

3、如果您投资了44枚代币，其他两人投资的数目分别是55枚、60枚，那么您将从公共账户口得到多少代币?

A、172
B、132
C、162

已回答，点击此处，进入下一步

Rule Comprehension Task:

To ensure you have understood instruction of the game, please answer the questions carefully. ”1. If you invest 5 tokens, and the other two people invest 55 and 60 tokens respectively, how many tokens will you receive from the public account?

A: 175; B: 110; C: 120;

2. If you invest 20 tokens, and the other two people invest 55 and 60 tokens respectively, how many tokens will you receive from the public account?

A：175; B: 170; C: 160;

3. If you invest 44 tokens, and the other two people invest 55 and 60 tokens respectively, how many tokens will you receive from the public account?”

A: 172; B: 132; C: 162;

I have answered these questions, click here to next step.

Page 7 Your role

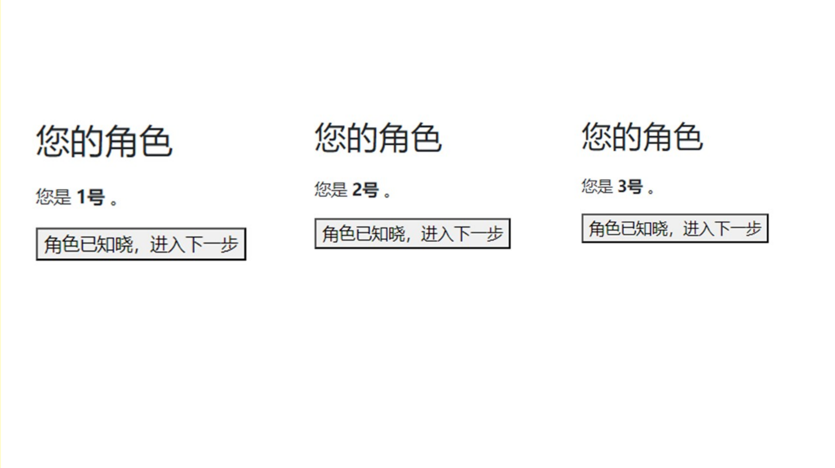

Your role:<br>You are Player 1.<br><br>I have understood the role, click here to next page.	Your role:<br>You are Player 2.<br><br>I have understood the role, click here to next page.	Your role:<br>You are Player 3.<br><br>I have understood the role, click here to next page.

Page 8 Your decision

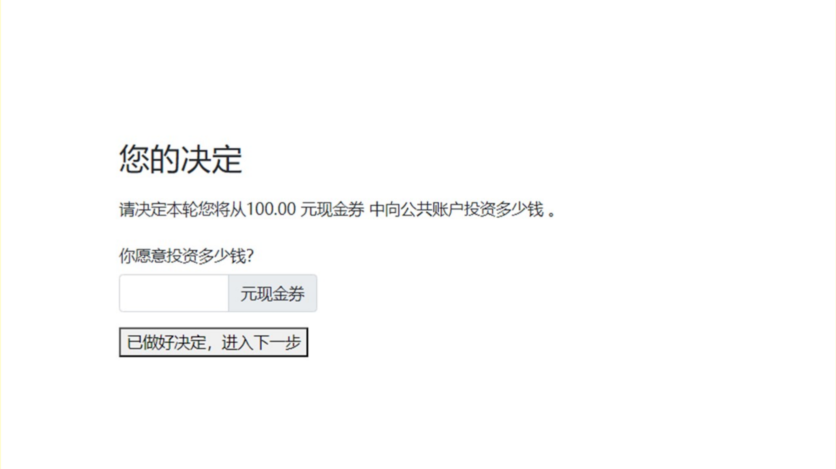

Please decide how many tokens you will decide to contribute from 100 in to public account.
How many tokens you are willing to invest
Click here to next page.

Page 9 waiting page

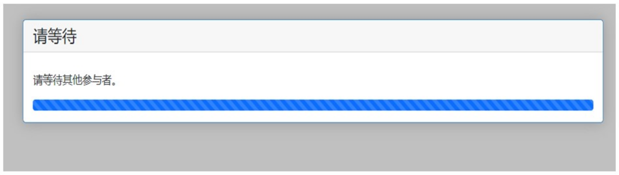

Waiting for Other participants to decide.

Page 10 Feedback

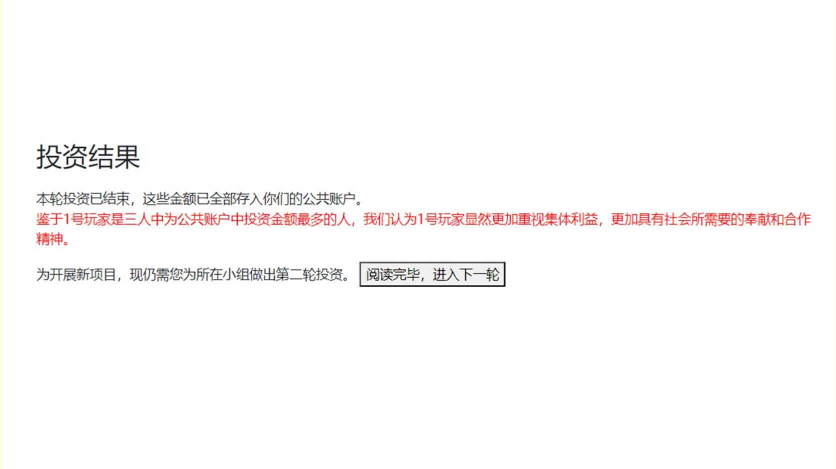

Investigation Outcome

This round of investment has ended, and these amounts have been fully deposited into your public account.

From this round of investment, it can be seen that Player 1 is a person who values collective interest and has a spirit of dedication.

For start a new project, now you need to investigate in a second round.

Click here to next page.

Page 11 Your decision

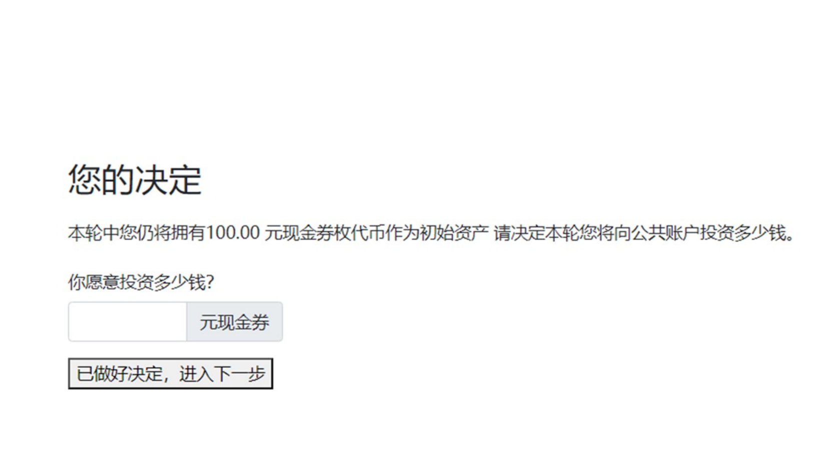

Please decide how many tokens you will decide to contribute from 100 in to public account.

How many tokens you are willing to invest

Click here to next page.

Page 12

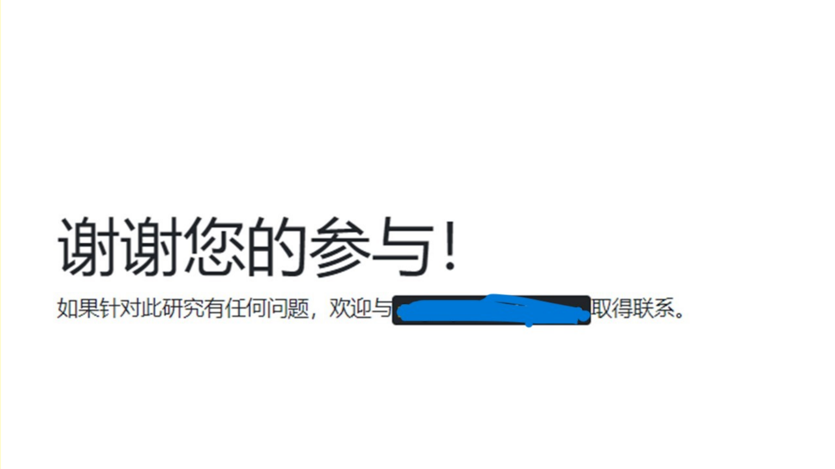

Thank you for your attention.

2 No Praise Condition

Page 1 Welcome Page

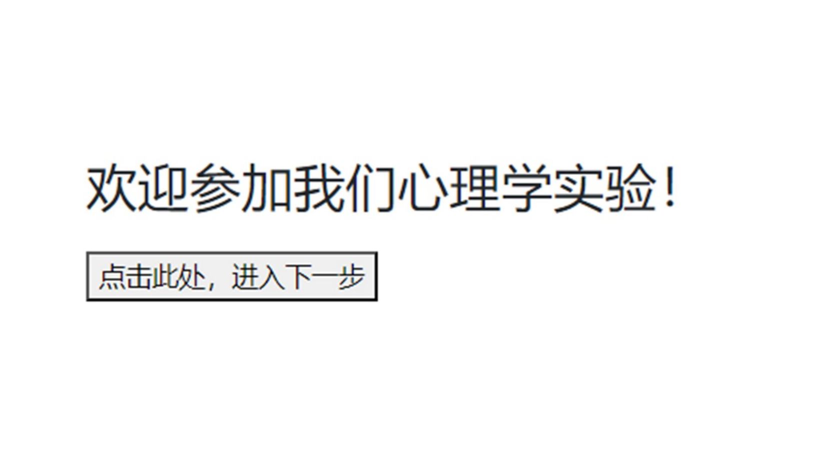

Welcome to attend our psychological experiment.
Click here to Next Page.

Page 2 Study Information Notice

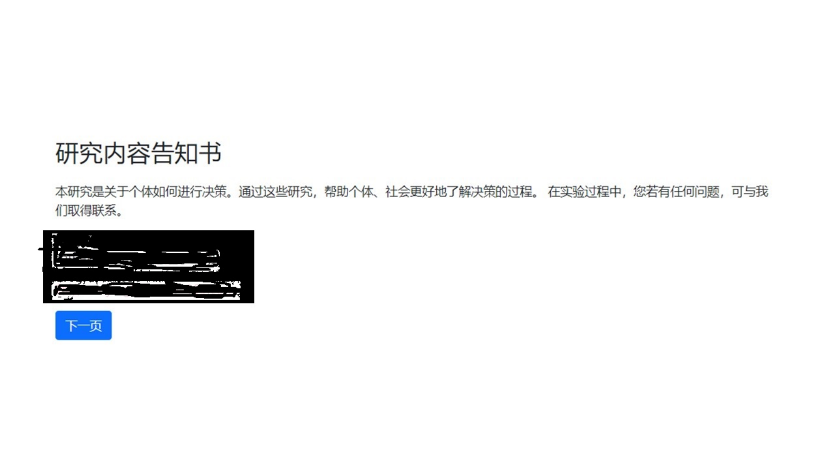

Study Information Notice

This study is about how to make decision. Through these studies, we are able to help individuals, society to understanding the process of decision making. During the progress of experiment, if you have any questions, please feel free to contact me.

Contact Information of Researchers. [cover for privacy issue.]

Page 3 Personal Information Collection

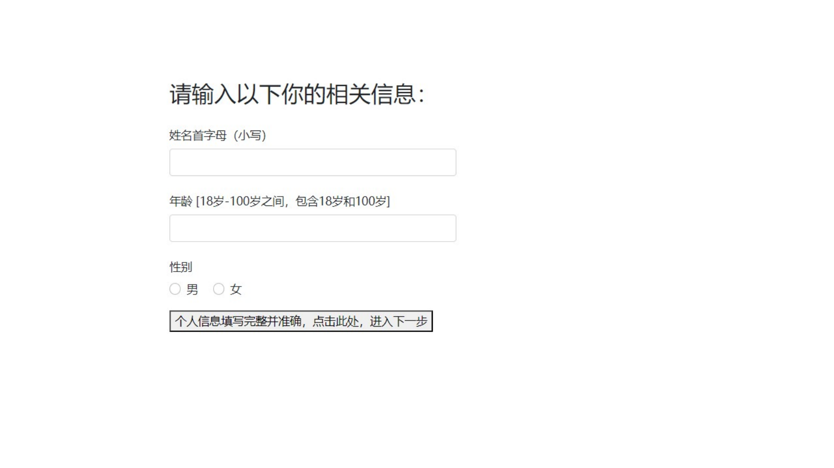

Your initial name (lower cases)

age: (18 to 100 years old)

gender: male; female

Click here to the next page.

Page 4 Experimental Instruction

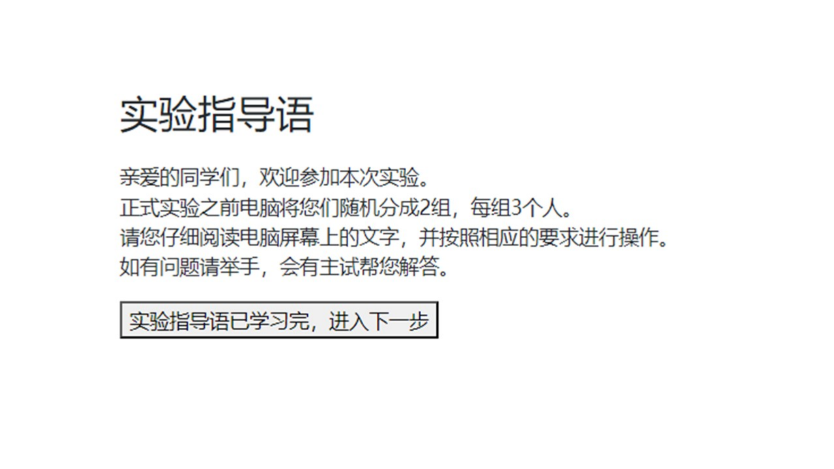

Experimental Instruction:
Welcome to our experiment. Before we start our experiment, you will be randomly into 2 groups, and each group will be assigned with three players. Please read carefully with the word in the screen, and operate according to the instruction. If you have any question, we will answer your question as detailed as we can.

Page 5 Instruction for the Game

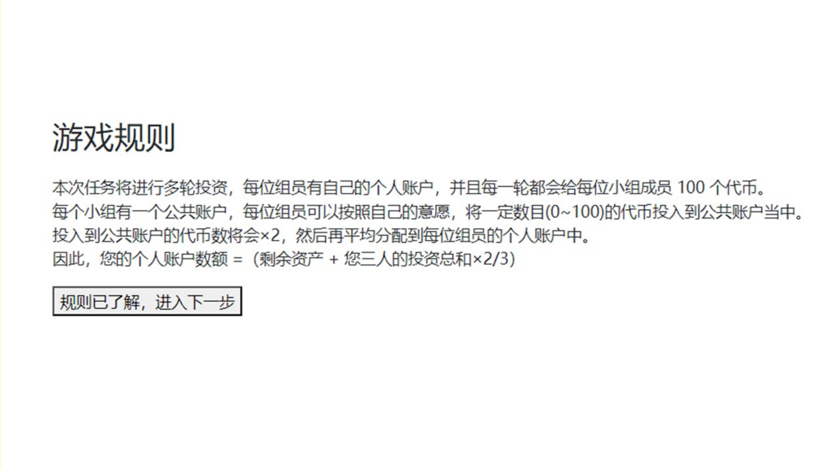

This task will involve multiple rounds of investment, with each team member having their own personal account, and each round, every team member will receive 100 tokens. Each group has a public account, and each member can invest a certain number X_i of tokens into the public account as they wish. $(0 <= X_i <= 100)$ The tokens invested in the public account will be doubled and then evenly distributed to each team member's personal account. The final amount in each person's personal account is: $(100 - X_i) + (X_i + Y) \times 2/3$.
I have understood the instruction, click here to next move.

Page 6 Rule Comprehension Task

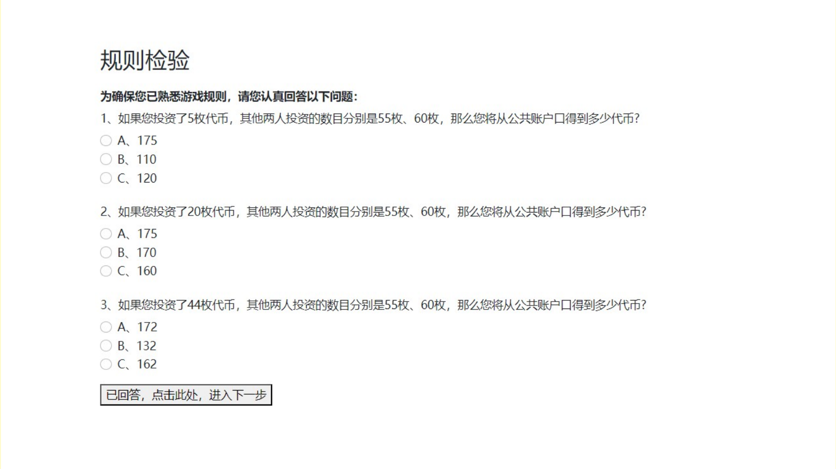
规则检验

为确保您已熟悉游戏规则，请您认真回答以下问题：

1、如果您投资了5枚代币，其他两人投资的数目分别是55枚、60枚，那么您将从公共账户口得到多少代币？

- A、175
- B、110
- C、120

2、如果您投资了20枚代币，其他两人投资的数目分别是55枚、60枚，那么您将从公共账户口得到多少代币？

- A、175
- B、170
- C、160

3、如果您投资了44枚代币，其他两人投资的数目分别是55枚、60枚，那么您将从公共账户口得到多少代币？

- A、172
- B、132
- C、162

已回答，点击此处，进入下一步

Rule Comprehension Task:

To ensure you have understood instruction of the game, please answer the questions carefully. ”1. If you invest 5 tokens, and the other two people invest 55 and 60 tokens respectively, how many tokens will you receive from the public account?

A: 175; B: 110; C: 120;

2. If you invest 20 tokens, and the other two people invest 55 and 60 tokens respectively, how many tokens will you receive from the public account?

A： 175; B: 170; C: 160;

3. If you invest 44 tokens, and the other two people invest 55 and 60 tokens respectively, how many tokens will you receive from the public account?”

A: 172; B: 132; C: 162;

I have answered these questions, click here to next step.

Page 7 Your role

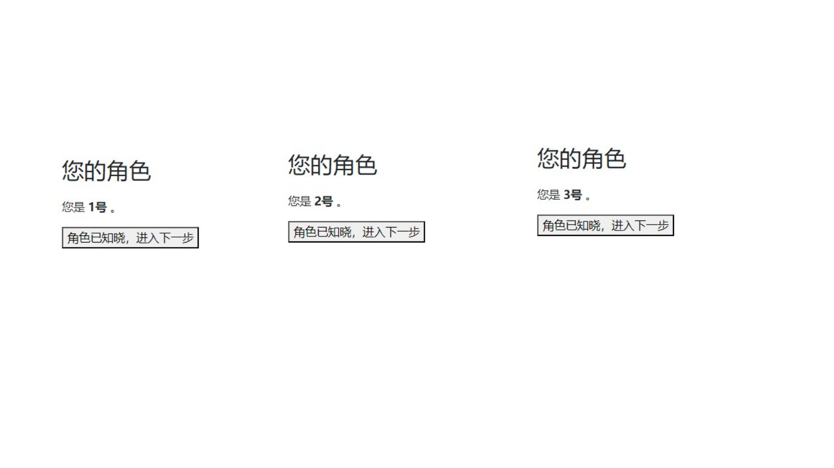

Your role:<br>You are Player 1.<br><br>I have understood the role, click here to next page.	Your role:<br>You are Player 2.<br><br>I have understood the role, click here to next page.	Your role:<br>You are Player 3.<br><br>I have understood the role, click here to next page.

Page 8 Your decision

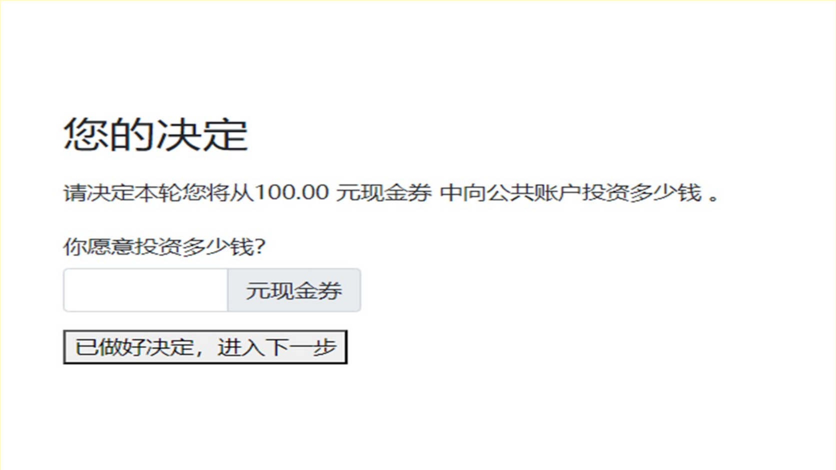

Please decide how many tokens you will decide to contribute from 100 in to public account.
How many tokens you are willing to invest
Click here to next page.

Page 9 waiting page

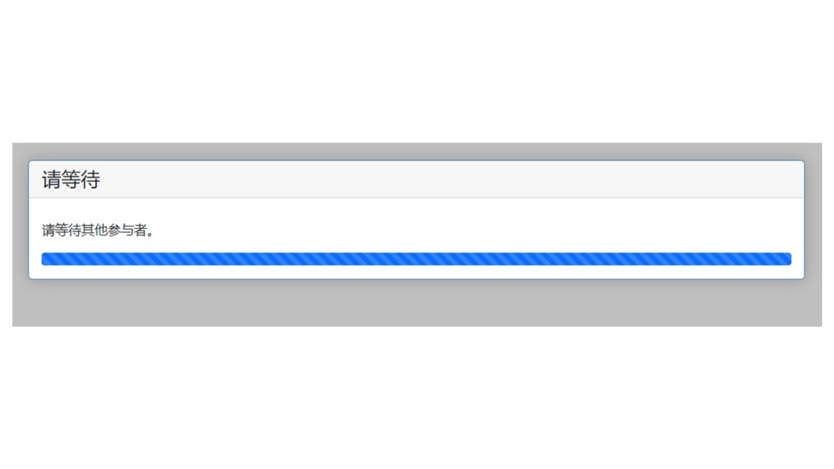

Waiting for Other participants to decide.

Page 10 Feedback

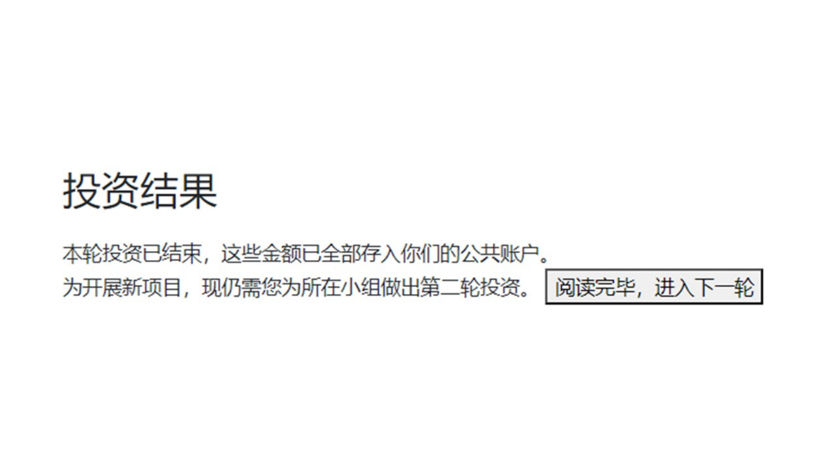

Investigation Outcome

This round of investment has ended, and these amounts have been fully deposited into your public account.

For start a new project, now you need to investigate in a second round.

Click here to next page.

Page 11 Your decision

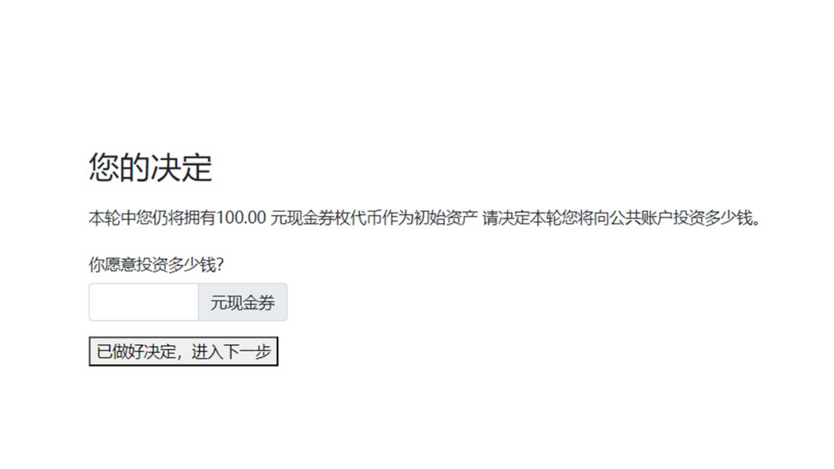

Please decide how many tokens you will decide to contribute from 100 in to public account.
How many tokens you are willing to invest
Click here to next page.

Page 12

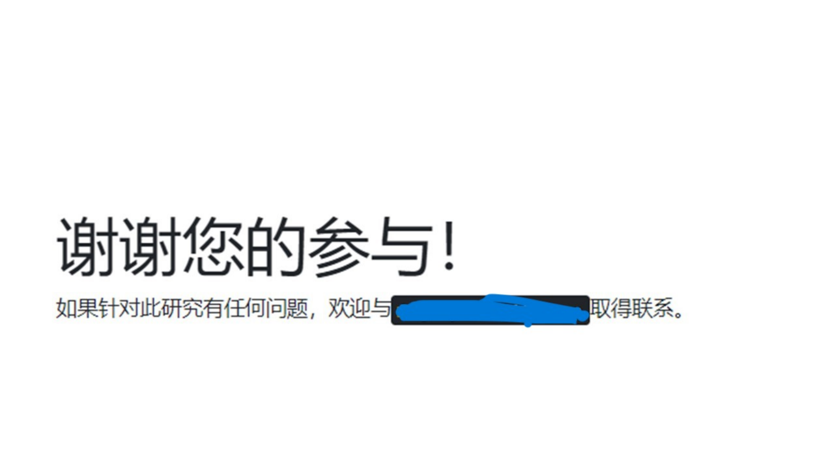

Thank you for your attention.
